# The Duties of the Dentist

**Published:** 1879-08

**Authors:** W. W. Ford

**Affiliations:** Macon, Ga.


### ARTICLE VIII.

### *The Duties of the Dentist.*

BY W. W. FORD, MACON, GA.

There are few, if any, of the callings of life that makes a greater demand upon both the mental and physical powers of man, than that of dentistry. It is a profession that not only employs and severely taxes *all* his mental faculties, but it taxes to their extreme limit every physical energy of which man is possessed; every muscle, ligature and fibre in his entire body is brought into constant action of the most trying nature while he is performing his professional duties.

The dentist who serves his patients faithfully and conscientiously all through the long, weary day, is, at its close, not only very tired in body, but his eyes and all his mental energies are completely exhausted. Therefore, dentists, as a class, do not live to be very old men, "and if by reason of strength" they should live to pass three score, they are, or have to be, laid upon the shelf, so far as their professional duties are concerned, for they are no longer physically capable of performing its most arduous and fatiguing duties.

There are no class of men in any of the learned professions that wear out so soon, or so completely as the dentist does. I claim, therefore, to make a dentist—that is a success—you must have *a whole man*, both mentally and physically, to make him out of; there is nothing belonging to him proper that you can dispense with in the make-up; you may then make only a piece of a dentist out of your whole man, but you cannot possibly make a whole dentist out of a piece of a man. He must have the physical development in body necessary to enable him to endure the great fatigue attending the faithful performance of his professional duties; he must have a correct, close-seeing and fine discriminating eye; he must have a skillfully trained ingenious hand, that is capable of the most delicate touch, not only in handling his patients, but also in handling his instruments and appliances in such a manner as to mould and form his work so as to make it not only the most beautiful and natural in appearance, but the most durable and lasting; he must have a clear mind, a close, discriminating intellect, a good sound judgment, to teach him when, where and how to operate and treat the varied cases that come before him; and above all, and over all, he must be honest, trustworthy and conscientiously faithful to do all that he can do for the redemption and salvation of the dental organs of his patients; he must be mild and gentle in his manner and deportment to his patrons, or those who visit his office; he must be easy and sympathizing in all cases where he has to inflict pain, letting his patients know that he feels and fully appreciates the fact that he is working upon live tissue and nerves; he must not be impatient, rude, uncouth, or unkind to, or with those that require his services. Thus he must deport himself, not for one day only, but every day, and every hour of the day, whether he feels like it or not, sick or well; if he is able to be on duty these things must be done, however trying the case may be; however simple or foolish his patient may act; however cross-grained and unwell, tired and weary, and worn he may feel, he must not let anything betray him into saying an impatient word or giving a cross look; the public demand that their dentist be always in a good humor, and be always fresh, smiling and ready for their most unreasonable demands at any time they may find it convenient to make them.

Now, if it does not take a whole man, and a very good one at that, to perform all these things, then I would like to know what part of his mind, soul or body it is that is left unemployed.

The duties of the dentist is the more trying and wearing upon the human organism because it is not strictly mental or manual labor alone; but is both of them together, taxing at the same time, every energy of the mind and body ; consequently, it is impossible for the body and the vital energies to bear such a strain for a very long time without giving away ; hence, there be few, if any dentists, that ever live to see old age.

I do not say that every dentist in this country is wearing himself out, either mentally or physically, in the performance of dental duties. I wish I could say more of them were than there are, because such service is absolutely necessary to reach that high standard of success and proficiency which we all desire to attain, and see attained by every member of the profession. Dentistry, to be a success, is a profession of work, struggle after struggle, combat after combat, never yielding the field, never giving up, but constantly pressing onward and forward ; no time to lay down your instruments and fold your hands, work whether you have a patient or not; if you have no patient's mouth to work in, work in and on your own head; be training your hand to do what you have failed to accomplish before in the efforts you have made; search diligently with all your mind for those obstruse facts which you have not yet mastered in connection with the hidden mysteries of the "human form divine." And this advice is not given to the young members alone, either, for I never have yet seen the dentist that was so wise, mature, experienced and skillful, but what there was something in either science or art connected with dentistry for him to learn. "Work," "work," "work," is the inexorable decree of our profession, and he that is not ready and willing to do it had better stop at once; he has been mistaken in selecting his calling; he will never succeed, and he had just as well quit, and no longer deceive himself and the public by trying or pretending to do something that he is too lazy to do.

A lazy, good dentist I never saw in all my life. I have seen lazy men claiming to be dentists, but it was all a mistake, they were not dentists, nay, not even a half dentist; such a man may get the name and float along through life perfectly content just to know enough to do more harm than two industrious, energetic, skillful operators can undo or repair. Such men have just energy enough to ruin teeth, and not enough to save one.

How important our duties are; how much we have to accomplish; how varied, and intricate, and difficult a great many of our operations are; how skillful, how ingenious, how careful, and particular, and conscientious we have to be to do our whole duty and succeed.

Then, I say, we have but little time and talents to spare. Let us, of Georgia, be up and doing, and let our highest and only motto be good work, true work, and not who can do the cheapest, and consequently, the poorest and meanest stuff that can be palmed off upon a credulous community, thereby disgracing yourself, and the profession that you claim to be an honorable member of.—*Dental Luminary.*

